# Prognosis of COVID-19 in patients with breast cancer: A protocol for systematic review and meta-analysis

**DOI:** 10.1097/MD.0000000000021487

**Published:** 2020-07-31

**Authors:** Zhijuan Sheng, Li Zhang, Xinlu Liu, Li Yuan, Fei Li, Dingmei Dai, Shuilin Wu, Jingzhi Yang

**Affiliations:** a Department of Galactophore, Gansu Provincial Cancer Hospital; b The Third Ward of Cardiovascular Clinical Medical Center, Affiliated Hospital of Gansu University of Chinese Medicine; c The First Clinical Medical College of Lanzhou University; d The Second Clinical Medical College of Lanzhou University; e School of Public Health, Lanzhou University; f Rehabilitation Center Hospital of Gansu Province, Lanzhou, China.

**Keywords:** breast cancer, COVID-19, meta-analysis, mortality, SARS-CoV-2, severity

## Abstract

**Background:**

Coronavirus disease 2019 (COVID-19) has become a pandemic in the world and posed a great threat to people's health. Several meta-analyses have indicated that many comorbidities were associated with increased risk of COVID-19 severity or mortality. The original report also showed that the mortality rate of COVID-19 in breast cancer patients is more dependent on comorbidities than previous radiation therapy or current anti-cancer therapy. However, no meta-analysis has focused on this aspect. This systematic review aims to assess whether breast cancer will increase the severity and mortality of patients infected with COVID-19 and to explore which factors that may affect the severity or mortality rate of breast cancer patients with COVID-19.

**Methods:**

We will search the PubMed, Embase, Web of Science, the Cochrane Central Register of Controlled Trials (CENTRAL), China National Knowledge Infrastructure (CNKI), Chinese Biomedical Literature Database (CBM), and Wanfang database from December 1, 2019 to June 30, 2020. Cohort studies comparing the disease severity and mortality of COVID-19 patients with and without breast cancer will be included. Two independent reviewers will assess the risk of bias of the included cohort studies using the modified Newcastle-Ottawa Scale. We will conduct meta-analyses to calculate the risk ratio (RR) and 95% confidence interval (95% CI) using the random-effects model with the Mantel-Haenszel method. The Grading of Recommendations Assessment, Development, and Evaluation (GRADE) approach will be used to rate the quality of the evidence.

**Results:**

The results of this study will be published in a peer-reviewed journal.

**Conclusion:**

This study will provide comprehensive evidence for medical staff to adopt effective treatment strategies for breast cancer patients during the COVID-19 pandemic.

**PROSPERO registration number:**

CRD42020188208.

## 1 Introduction

In December 2019, a novel coronavirus named severe acute respiratory syndrome coronavirus 2 (SARS-CoV-2) caused the coronavirus disease 2019 (COVID-19), which has become a pandemic in the world and posed a great threat to people's health.^[1–3]^ As of June 16, 2020, 7941791 COVID-19 cases have been reported worldwide and 434,796 infected cases have died.^[4]^ Currently, there is no specific drug for the treatment of COVID-19. However, empirical evidence showed that traditional Chinese medicines such as Lianhua Qingwen granule and Shufeng Jiedu Capsules have a better effect on COVID-19.^[5–7]^

Several meta-analyses have indicated that many comorbidities, including cerebrovascular disease, cardiovascular disease, autoimmune disease, diabetes mellitus, hypertension, chronic pulmonary disease, immunosuppression, immunodeficiency, and liver disease, were associated with increased risk of COVID-19 severity or mortality.^[8–13]^ Previous meta-analyses also suggested that patients with a history of or active malignancy might be at increased risk of developing severe COVID-19 disease or mortality.^[14,15]^ However, these meta-analyses did not evaluate which types of cancer are more associated with COVID-19 progression and prognosis. Breast cancer is the most frequently diagnosed malignancy and the most common cause of cancer-related mortality among women in 2018.^[16]^ An original report showed that the mortality rate of COVID-19 in breast cancer patients is more dependent on comorbidities than previous radiation therapy or current anti-cancer therapy.^[17]^ However, these results have not been tested in a meta-analysis. Well-conducted systematic reviews and meta-analyses can provide the highest level of evidence for clinical practice.^[18,19]^ Therefore, we will perform a systematic review and meta-analysis to assess whether breast cancer will increase the severity and mortality of patients infected with COVID-19 and to explore which factors that may affect the severity or mortality rate of breast cancer patients with COVID-19.

## 2 Methods

This meta-analysis will be conducted and reported according to the Preferred Reporting Items for Systematic Reviews and Meta-Analyses (PRISMA) statement.^[20]^ The protocol of this study has been registered on the International Prospective Register of Systematic Reviews (PROSPERO, CRD42020188208).

### 2.1 Search strategy

We will search the PubMed, Embase, Web of Science, the Cochrane Central Register of Controlled Trials (CENTRAL), China National Knowledge Infrastructure (CNKI), Chinese Biomedical Literature Database (CBM), and Wanfang database from December 1, 2019 to June 30, 2020. To identify additional potentially eligible studies, we will also manually search the reference lists of eligible studies and relevant systematic reviews. Search terms we will use include “coronavirus disease-19,” “COVID-19,” “novel corona virus,” “new coronavirus,” “2019 novel coronavirus,” “2019-nCoV,” “novel coronavirus,” “nCoV-2019,” “novel coronavirus pneumonia,” “coronavirus disease 2019,” “severe acute respiratory syndrome coronavirus 2,” “SARS-CoV-2,” “breast cancer,” “breast neoplasm,” “breast tumor,” “phyllodes tumor,” “intraductal carcinoma,” “lobular carcinoma,” “clinical characteristic,” “clinical feature,” “risk factor,” “comorbidities,” and “prognosis.” The detailed search strategy of PubMed is presented in Table 1.

**Table 1:**
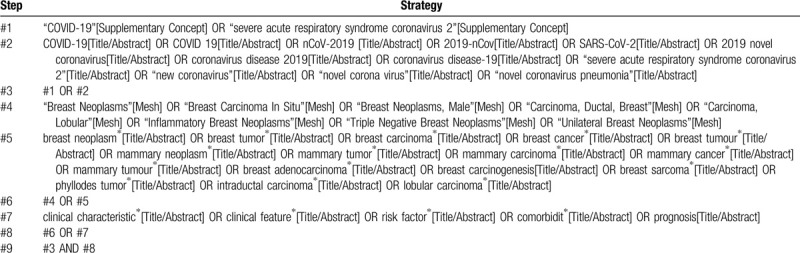
Search strategy of PubMed.

### 2.2 Inclusion criteria and exclusion criteria

We will include cohort studies that meet the following criteria:

1. patients enrolled in the study had a laboratory-confirmed diagnosis of COVID-19;
2. the study compared the disease severity and mortality of COVID-19 patients with and without breast cancer;
3. published in English or Chinese language;
4. studies included a sample size of larger than 10.

We will exclude studies with following characteristics:

1. studies included suspected cases;
2. studies compared the prevalence of cancer between severe and non-severe patients or between survivors and non-survivors without providing specific data on breast cancer;
3. animal studies, letters, comments, abstracts, editorials, and reviews.

### 2.3 Outcomes

The primary outcome is the association between breast cancer and the severity of patients with COVID-19. The secondary outcome is the association between breast cancer and COVID-19 mortality.

### 2.4 Study selection

We will use Endnote X8 (Thomson Reuters (Scientific) LLC Philadelphia, PA) software to manage the identified records and remove duplicates. Two authors will independently screen the titles and abstracts to determine which studies should be further evaluated. Any potential studies will be retrieved for full-texts to determine the final eligibility according to the inclusion and exclusion criteria. Any discrepancies will be resolved through consensus or recourse to a third author. If we identify studies with overlapping data, the study with a larger sample size will be used.

### 2.5 Data extraction

We will develop a data extraction form using Excel 2016 (Microsoft Corp, Redmond, WA, www.microsoft.com) to abstract information from included studies. Two reviewers will independently conduct data extraction and disagreements will be resolved by discussion. The detailed information will include first author, year of publication, country of the first and corresponding author, journal name, publication language, study setting, study period, duration of follow-up; age and sex of patients, sample size, number of breast cancer patients; number of severe cases, non-severe cases, survivors, and non-survivors. The COVID-19 definition of severe respiratory infection from the World Health Organization will be used to define disease severity: fever or suspected respiratory infection, plus 1 of the following: respiratory rate > 30 breaths/min, peripheral oxygen saturation (SpO2) ≤ 93% on room air, or severe respiratory distress.^[21,22]^

### 2.6 Assessment of risk of bias

The quality of included cohort studies will be assessed using the modified Newcastle-Ottawa Scale (NOS) for comparative cohort studies.^[23]^ This tool assesses the sources of bias in the selection of participants, temporality of outcome relative to exposure, measurement of outcome, exposure and prognostic factors, balance in prognostic factors, balance in concomitant therapy across groups, and completeness of follow-up.^[24]^ Two independently reviewers will judge each item as “definitely yes,” “probably yes,” “probably no,” or “definitely no.” Disagreements will be resolved by discussion with a third reviewer.

### 2.7 Statistical analysis

We will conduct meta-analyses to calculate the risk ratio (RR) and 95% confidence interval (95% CI) to estimate the prevalence of the severe disease and non-survivors in COVID-19 patients with or without breast cancer. Considering the heterogeneity between studies, we will use the random-effects model with the Mantel–Haenszel method. Heterogeneity will be evaluated using the Chi^2^ test and the I^2^ statistic. The I^2^ statistics of 25%, 50%, and 75% represent low, moderate, and high heterogeneity, respectively.

### 2.8 Subgroup analysis

Subgroup analysis will be performed for the primary outcome and secondary outcome between different countries.

### 2.9 Sensitivity analysis

Sensitivity analyses will be conducted by sequentially excluding one study at a time, by excluding small sample studies, or by removing low-quality studies to check whether the results are robust.

### 2.10 Meta-regression analysis

If the necessary data are available, we will perform meta-regression analyses to assess whether the publication languages, duration of breast cancer, other comorbidities, or treatment regimen will affect the results.

### 2.11 Publication bias

We will adopt the Egger's test and funnel plot to assess the potential publication bias for outcomes with studies more than nine.^[25]^

### 2.12 Quality of evidence

We will use the Grading of Recommendations Assessment, Development, and Evaluation (GRADE) approach to evaluate the quality of the evidence.^[26]^ The GRADE includes five considerations: risk of bias assessment, inconsistency, indirectness, imprecision, and publication bias.^[27,28]^ The quality of evidence for each outcome will be rated as high, moderate, low, or very low by two independent review authors. We will present the level of evidence in the Summary of Findings table.

## 3 Discussion

Reliable assessment of breast cancer and COVID-19 disease progression and prognosis is critical to ensure comprehensive global preventive and treatment strategies for breast cancer patients under the COVID-19 epidemic. To be the best of our knowledge, this study will be the first meta-analysis that focuses on the association between breast cancer and COVID-19 severity and mortality. We hope that our results will provide comprehensive evidence for medical staff to adopt effective treatment strategies for breast cancer patients and to promote the development of high-quality evidence-based guidelines to guide management, diagnosis, and treatment of breast cancer patients during the COVID-19 pandemic.

## Author contributions

**Conceptualization:** Zhijuan Sheng, Li Zhang, Jingzhi Yang.

**Funding acquisition:** Zhijuan Sheng.

**Investigation:** Zhijuan Sheng, Xinlu Liu, Li Yuan, Fei Li, Dingmei Dai, Shuilin Wu.

**Methodology:** Zhijuan Sheng, Li Zhang, Jingzhi Yang.

**Project administration:** Jingzhi Yang.

**Resources:** Zhijuan Sheng, Li Zhang, Xinlu Liu, Li Yuan, Fei Li.

**Supervision:** Jingzhi Yang.

**Validation:** Jingzhi Yang.

**Visualization:** Li Zhang, Xinlu Liu, Li Yuan, Fei Li.

**Writing – original draft:** Zhijuan Sheng, Li Zhang, Jingzhi Yang.

**Writing – review & editing:** Zhijuan Sheng, Li Zhang, Jingzhi Yang.
